# Pre-exposure prophylaxis makes it possible to better live one’s sexuality and guide men who have sex with men towards a responsible approach to their health: a phenomenological qualitative study about primary motivations for PrEP

**DOI:** 10.1186/s12981-020-00327-7

**Published:** 2021-01-07

**Authors:** Marie Bistoquet, Alain Makinson, Vincent Tribout, Cyril Perrollaz, Gérard Bourrel, Jacques Reynes, Agnès Oude Engberink

**Affiliations:** 1 Département Des Maladies Infectieuses, Centre Hospitalier Universitaire, Montpellier, France; 2 UMI 233/INSERM U1175, Université de Montpellier, Montpellier, France; 3 Centre Gratuit, D’information, de Dépistage Et de Diagnostic Des Infections Sexuellement Transmissible, Centre Hospitalier Universitaire, Montpellier, France; 4 Département Universitaire de Médecine Générale, Université de Montpellier, Montpellier, France; 5 Institut Desbret D’Épidémiologie Et de Santé Publique, Université de Montpellier, Montpellier, France; 6 Maison de Santé Pluriprofessionnelle Universitaire Avicenne, 2 rue IBN Sinaï Dit Avicenne, 66330 Cabestany, France

**Keywords:** Pre-exposure prophylaxis, HIV prevention, Quality of life, Qualitative research, Phenomenology

## Abstract

**Background:**

Pre-exposure prophylaxis (PrEP) for HIV is instrumental in the prevention of HIV for HIV-uninfected persons, by drastically reducing the risk of acquisition in the case of high-risk exposures. Despite its demonstrated efficacy, it remained under-prescribed in France until 2018. The principal aim of this study was to understand the motivations of Men who have Sex with Men (MSM) who started using PrEP in Montpellier, France.

**Methods:**

A phenomenological study was undertaken, using semi-structured interviews with twelve participants attending the University Hospital of Montpellier for PrEP. Interviews were analysed by means of triangulation up to the point of theoretical saturation, using a semio-pragmatic method.

**Results:**

Fear of HIV infection, personalised regular follow-up, and the wish to take care of one’s health were the primary motivational factors. PrEP allows for a better sexual life restoring a sense of freedom despite the risks of STI, deemed manageable by PrEPers. PrEP does not modify long-term risk-taking behaviours but helps them better live their own sexuality and guides them towards a responsible approach to sexuality. Unclear information on PrEP, delivered by their family doctor, public campaigns or the media, leads to misrepresentations or negative social representation, including within the MSM community, which may delay its implementation.

**Conclusions:**

Fear of HIV infection and the benefits of regular medical follow-up to take care of one’s health were motivational factors of importance for the use of PrEP by MSM in this study. PrEP transforms all existential dimensions of their lived experience, improving sexual identity and happiness. There is a need to improve professional awareness of the effectiveness of PrEP and to develop a patient centered approach, to disseminate information more widely to the general public and among MSM to reduce stigmatisation.

## Background

In 2018, approximately 37.9 million people were living with Human Immunodeficiency Virus (HIV) globally [1]. In 2018, 6,200 people have been diagnosed positive for HIV in France [2]. Since 2011, the annual incidence remained stable, except for a 7% decrease in 2018. The incidence of HIV is high among men who have sex with men (MSM) and this is the group at highest risk of acquiring the infection [3].

Pre-exposure prophylaxis for HIV (PrEP) is a biomedical intervention to prevent HIV infection, available in the United States since 2012 [4] and in France since 2016 [5]. It is aimed at individuals not infected by HIV, in order to reduce the risk of infection in case of high risk sexual practices. PrEP is effective, with a relative reduction of HIV transmission in different studies of more than 90% if good adherence is ensured [6–10]. In France it can be used daily (one tablet per day) or on demand (two tablets two hours before sexual activity then one tablet 24 and 48 h after). However, PrEP has remained under-used in France [11] with studies in other countries highlighting general public, MSM and practitioners’ lack of knowledge and criticism of this prophylaxis as factors for non-prescription [12, 13]. Several questions were raised from the 2012 International Symposium on HIV & Emerging Infectious Diseases (ISHEID) [14]: is high adherence for daily PrEP achievable? Is there a risk of change in behavior that could offset the benefit of PrEP?

Research has been published on the motivations of those undertaking PrEP [15]. According to Golub et al. [16] in a computerized survey the most highly endorsed facilitator was free access to PrEP, regular HIV testing, sexual health care/monitoring, and access to one-on-one counseling. In a cross sectional study, age, education and intimacy motivations for condomless sex were significantly associated with PrEP adoption intention [17]. Some barriers were highlighted in a systematic review [18]: lack of knowledge, stigma, risk taking and adherence for patients, lack of knowledge of treatment and protocols, fear of resistance and behavioural change for care providers.

A review of the literature on the values and preferences regarding the utilisation of PrEP in the prevention of HIV concluded that a better understanding of the perception of users would improve utilisation of PrEP in different countries [19].

The aim of this study was to understand the motivations of MSM PrEP users in Montpellier, through the use of a phenomenological approach centred on their lived experience.

## Methods

### Recruitment

Volunteers were sequentially recruited in the Department of Infectious and Tropical Diseases and the sexually transmitted infection (STI) Screening Centre at Montpellier Hospital. Each person presenting for a PrEP consultation was invited by the medical physician to participate and informed consent was sought.

Volunteers contacted the medical resident responsible for the interviews (MB), via a dedicated email address. All interviews took place at the Department of Infectious and Tropical Diseases of the Montpellier University Hospital, using a dedicated office, and in the sole presence of the resident.

The sampling followed the principle of a purposive sample [20], in which participants varied according to the following characteristics: socio-professional level, age, place of consultation, use of substances (recreational and or illicit), and duration of PrEP. In order to obtain ‘information-rich’ cases, the recruited volunteers had varied characteristic profiles. Participants’ opinions regarding PrEP were not known before the interviews. The size of the sample was not established in advance, but instead data adequacy was determined from data emergence and saturation on material analysis.

### Collection of material

The material for analysis was gathered through the use of in-depth interviews, using an interview guide with phenomenologically-oriented questions centred on the participant’s lived experiences (Table 1). The investigator received prior training enabling him to prepare his interviews to facilitate the authenticity of the responses, taking into account his involvement as a researcher. The interviews took place between February and June 2018 and were audio-recorded then transcribed verbatim. The identifying features of all participants were anonymised after signing a consent form for the analysis and publication of data. The participants were not known to the researcher before the interviews took place. Each verbatim transcript received a number to ensure anonymity.

**Table 1:** Interview guide

1/ To begin with, I’d like to know how you first learned about PrEP? What was your perception of PrEP before coming for your first consultation? What were your expectations of PrEP prior to the first consultation?
2/ What finally motivated you to come for a consultation with Dr (official PrEP doctor’s name) and to begin this process? What was your experience of this consultation? What did you understand?
3/ Since you have started PrEP, what changes have you seen in your life? (these could include personal, intimate, as well as family and professional experiences)
4/ How do you perceive HIV now? What does this illness mean to you ?
5/ Today, what is PrEP for you? What constraints or restrictions do you feel in taking this prophylaxis? What advice would you give to friends or those you know, who are interested in PrEP?

Ethical approval for this research was granted by a regional Committee for the Protection of Persons in accordance with the current regulation (No IDRCB 2017-A03467-46, CPP 18013).

### Phenomenological pragmatic analysis of the interview material

We used a semiopragmatic phenomenological approach in accordance with our objective of exploring the lived experience of MSM. It is a specific phenomenological approach based on C.S. Peirce ‘s theories and is a descriptive method for categorizing lived experiences recorded in interview transcripts. The first steps of this analysis (Table 2) were performed according to a constant comparison process [21] to build the categories, completed by a semiopragmatical data interpretation procedure inspired by C.S. Peirce [22]. In this method, the analyst takes into account all the semiotic elements of a text, including linguistic and contextual clues. First, empirical categories emerge by constant comparison. Secondly semiopragmatical analysis allowed the logical ordering of these empirical categories according to Peirce’s theory of signs. Typically, as a result of this ordering, the conceptually densest category (i.e., of the highest level in the hierarchy of signs) commands the meaning of the phenomenon at play. The steps of the method used are summarised in Table 2 [23]

**Table 2:** Steps of a pragmatic phenomenological analysis

Word by word transcription of recordings (verbatim)
A reading using a floating attention, followed by a focussed reading
Extracting signifying units from the text and grouping these units by themes
Collating textual and contextual meaningful semiotic elements and their semio-pragmatic characterisation
A first categorisation through a regrouping of these semiotic elements and of the signifying units in accordance with the research question
Enriching the categories by continuing comparison until theoretical saturation is reached
Placing the emerging categories in logical order and reducing them and their properties in order to model the ensemble in integrative semio-pragmatic statements

### Reliability criteria

The work is reported in conformity with the COREQ criteria [24]. The validity of coherence [25] has been respected by the congruence between the research objective and the choice and conduct of the methodological steps of the research, and making these rigorously explicit. The first interview was treated as a trial. The sole interviewer, a medical resident, had received prior training in the process of phenomenological reformulation to perform in-depth interviewing. When necessary, after certain interviews, the investigator took field notes to distance himself from his feelings. The triangulation of researchers was obtained between two experts in qualitative research (GB, AOB) and the interviewer, who pooled their analyses. No further interviews were performed once saturation of the material was reached. No participant declined release of the results of his interview. A semio-pragmatic analysis is a qualitative research method that proposes the logical ordering of empirical data according to their semiotic characterisation, enabling the reduction of bias of interpretation without compromising the emergence of new results [26].

The study (collection, transcription, analysis) was carried out in French. The first version of the article was written in French and then translated for dissemination of the results to a broader community.

## Results

### Participant characteristics

Each of the twelve interviews lasted between 35 and 45 min. Participants described their personal lived experience freely, without restraint. Their ages ranged from 28 to 52 years. Other characteristics are summarised in Table 3.

**Table 3:** Participant characteristics

	Age	Education level	Profession	Use of substances	PrEP status
E1	30	Bac* + 5	Socio-cultural animator	0	Daily
E2	49	CAP**	Unemployed	THC	Daily
E3	30	Bac + 5	Physiotherapist	Tobacco MDMA	Daily
E4	45	Bac + 2	Self-employed	0	Daily
E5	28	Bac + 9	Medical doctor	Tobacco Alcohol	event driven
E6	46	Bac + 5	Teacher	Alcohol	event driven
E7	38	Bac + 4	Company director	Tobacco	Daily
E8	52	Bac + 9	Unemployed	0	Daily
E9	41	Bac	Computer programmer	Alcohol	Daily
E10	31	Bac + 9	Veterinary surgeon	Alcohol E cigarette	Daily
E11	28	Bac + 2	Building draftsperson	Alcohol	event driven
E12	49	Bac + 5	Project manager	Alcohol	event driven

Note: Participants are numbered according to the order of inclusion in the study

* Bac (*Baccalaureate*) is the end of secondary school qualification

** CAP is equivalent to a trades certificate

*** THC: Tétrahydrocannabinol

**** MDMA: 3,4-Methyl enedioxy methamphetamine

### Semio-pragmatic phenomenological analysis

Theoretical saturation is the result of the data analysis process as each interview is carried out. This is a continuous comparison procedure that allows the emerging categories to be enriched to the highest density. The density of a phenomenological category represents the level of information it contains about the phenomenon being studied. When the main categories are saturated, this means that there is no need for further interviews.

Five phenomenological statements emerged from the analysis.

### The trivialisation of HIV, leading to a decrease in prevention, contrasts with the persistent fear of acquiring HIV and a stigmatising representation of the infection

Interviewees highlighted a decrease in protective behaviors (particularly condom use): ‘*we meet more and more people who refuse to use a condom’* E8. This observation was all the more present among the younger generation and was accompanied by a change in the perception of HIV: ‘*I knew a time when one would die from AIDS […] and it became a chronic illness’* E12; ‘*we are not in the same era, there is a different state of mind’* E12; ‘*anyway, it’s less dangerous’* E6.

For older interviewees, having had their first sexual experiences before the era of effective antiretroviral combinations, anxiety persisted regarding the possible transmission of the virus. This fear was embodied in their lived experience: ‘*for me, I still have the image of skeletal people with Kaposi marks, that I’ve seen my whole life*’ E2.

This era prior to antiretroviral combinations was experienced as traumatic for some participants and affected their current sexual behaviour considerably: ‘*The first image that I had of homosexuality was linked to death’* E7.

Finally, the stigmatised perception of HIV remained present: ‘*I still have this image of a terrible illness, that mustn’t be caught at any cost […] I’d really prefer to have a cancer than to catch this disease because, there it is, there’s everything behind it, everything that surrounds the thing…’* E4.

### Unclear information on PrEP delivered by their family doctor, public campaigns or the media leads to misrepresentations or a negative social representation, including within the MSM community, which may delay its implementation

Most of the interviewees had first heard about PrEP from the televised media or through social networks: ‘*On the tv news.* After *going out, in some places, I saw that there were small leaflets, but for a long time I didn’t look at them anymore because it was always about the same thing’* E6; ‘*social networks first’* E8.

Those who discovered PrEP via a prevention campaign said that they did not feel concerned by the content of a prevention campaign about PrEP and had not understood the message (‘*I didn’t have enough information, and that made me fearful, and I believed more that it was a treatment against AIDS’* E2) or not feeling concerned (‘*It was about protecting me from something, but it’s an open door for all the STDs […] I didn’t feel very concerned about my sexuality’* E7).

Others tried to inform themselves through their family doctor, without more clear understanding (*my GP spoke to me about post-exposure prophylaxis (PEP) and therefore it was very vague*’ E1), or indeed even feeling judged ‘*when I spoke to her about PrEP, she didn’t know. And when I explained to her what it was, she had a prejudiced reaction that shocked me.’* E4.

Almost all of those interviewed said they found clearer responses to their questions through social media (Facebook page on PrEP, through word of mouth (‘*people I met who were on PrEP*’ E8), through the Grinder meeting app (‘*so I'm on grinder, I think that's where I heard about it first’* E9), or through information sites such as AIDES or sida-info service.

The first representations of PrEP, coming from the media, were rather negative as well as those coming from the general population (‘*in the minds of people, it’s very stereotyping’* E3) as well as in the MSM community (‘*Negative remarks, even discrimination in relation to PrEPers, so there it is, a representation … of debauch, inevitably of filth […] considering them almost as having the plague*’ E4).

Approximately half of those interviewed did not consider their practices as very high-risk and therefore did not know whether they would benefit from PrEP: ‘*it was designed for people who have high-risk practices […] I’m not certain that I would benefit from it’* E4; ‘*for people who don’t want to use condoms’* E6.

Although many were open about their homosexuality socially, they preferred not to reveal their PrEP status for fear of being perceived as having an unrestrained sexuality (‘*you don’t talk about it, nor do you talk about it to your family’* E4, E6) or of being a carrier of disease by their potential partners (‘*it’s always viewed as […] people who take risks, who have an unrestrained sexuality or who do anything’* E3.

### Fear of acquiring HIV, benefitting from regular, personalised medical follow-up, and the wish to take care of one’s health, are the principal motivational vectors for PrEP

The primary motivation of almost all of those interviewed was the protection PrEP offers against HIV and of the anxiety of being infected by HIV (‘*the fear, every time of being infected with HIV’* E2, ‘*my primary concern is above all not to catch the disease’* E12).

The second motivation that was highlighted for half of those interviewed concerned the regular, personalised and rigorous follow-up that is available with PrEP. They strongly appreciated being listened to without prejudice, and also appreciated the regular HIV and STD screening: ‘*serious […] well framed […] reassuring […] good supports […] comforting*’ E10. The CeGIDD [Screening Centre] was considered to be a referral centre, outside the usual path of care, where interviewees felt free to discuss: ‘*reassuring, good follow-up, you can discuss everything’* E10.

The delay for reflection left between the first two appointments before starting PrEP played a large role in their decision and their compliance: ‘*At first I didn't want to take it […] he offered it to me, my opinion has changed a little, I said to myself if it's effective…’ E9, ‘it forces people to think, it's not necessarily worse*’ E8. ‘*it’s different for each person, each case is personal*’ E9.

This regular follow-up also gave them the feeling of being better monitored and taking responsibility for their own care: ‘*we take care of our health, we are screened more often and more thoroughly’* E4; ‘*the PrEP has changed my life in the way that I take a bit more care of my health*’ E2. ‘*Taking care of oneself […] it’s more on the side that I feel like taking care of myself, of my health’* E3; ‘*it’s a complementary comfort but it’s not a permission to do whatever one wants’* E1; ‘*it’s an insurance, […] a comfort, […] a lifeline’* E12.

### PrEP allows for a better sexual life restoring a sense of freedom despite the risks of STI, deemed manageable by PrEPers

The PrEP experience was felt to be very positive: ‘*it’s a great thing, a very positive image, a real bonus’* E12; ‘*it’s magic’* E4.

PrEP allowed them to be more appeased, to be sexually more fulfilled, freer and more accepting of themselves: ‘*we no longer have shame waiting for the tests, we’re more serene*’ E9; ‘*it’s the first time that I live my sexuality so well’* E6.

The counterpoint to this feeling of freedom is the fear of adverse events that sometimes hindered PrEP initiation. Three declared having experienced adverse events (digestive problems): ‘*it’s a medicine to take every day, it’s pretty heavy*’ E1; ‘*I’m fearful of the side effects on the body, it’s still a strong medication’* E12.

### PrEP does not modify long term risk-taking behaviours but appeases the lived experience and guides them towards a responsible approach to sexuality

Five participants verbalised spontaneously not having changed their sexual behaviour following a course of PrEP. They perceived a feeling of freedom and well-being: ‘*no, it didn’t lead to any particular change, simply a freedom from doubt*’ E1.

Increased sexual risk-taking was the case, however, for three participants who emphasised a strong increase in risk-taking shortly after the beginning of PrEP combined with a period of reducing the use other preventive strategies (‘*there was a phase, just at the start of taking the PrEP, of a certain sense of all-powerfulness, one is not invulnerable, but almost’* E7).

This ‘UP phase’ somehow remained transient in each case. Each participant said that they were caught again, at one time or another, by fear of other STDs, or by a feeling of guilt seriously disrupting their self-esteem and their values. They returned to their old practices: ‘*unrestrained sexuality, it’s not something that I value personally […] I prefer to stay in the frame of safe-sex’* E12.

In contrast, for some, PrEP paved the way towards a project of being self-responsible (‘*the PrEP has changed my life’* E2), and a new sense of self-esteem, resulting in a better acceptance of their sexuality: ‘*it must not make us forget our other objectives in life, the professional side’* E12.

For some interviewees, PrEP triggered a willingness to advocate accurate information about PrEP and thus limit stigmatisation of PrePers,: ‘*I encourage people to come for screening’* E1; ‘*I try to start a conversation that demystifies the prejudices*’ E4; ‘*there is a gap in understanding and information’* E10.

## Discussion

Through genuine exchanges, our qualitative approach enabled a better understanding of the perceptions and motivations of MSM undertaking PrEP.

In the context of decreased HIV prevention and an associated increase in STD incidence [27, 28], the fear of acquiring HIV was present for nearly all participants. This fear was the main motivation behind PrEP uptake. The second motivation was the chance to have regular and personalised medical monitoring. This result confirmed that of a Canadian study showing strong attendance at follow-up visits [29]. This notion of consultations dedicated to sexual health has been observed previously [16], and is linked to a desire to take care of their own health.

Although PrEP was not seen as a means to take additional risks, it nevertheless caused a transient decrease in other means of protection, and a possibility of exceeding limits for some interviewees as in Scott's study [30]. This feeling of ‘*invulnerability’* temporarily diminished their vigilance with regard to other preventive measures in our study, but interviewees were quickly caught by a negative self-esteem and feeling of guilt driving them to resume their original behaviours, as evidenced in other studies [31, 32]. Kenyon’s study findings about chemsex provide further evidence of the importance of asking MSM clients about the use of psychoactive substances during consultations and tailoring interventions such as pre exposure prophylaxis, more frequent STI screening and substance abuse counseling accordingly [33].

The transcripts are marked by linguistic indices connoting ‘*a better life’*, with PrEP transforming all existential dimensions of their lived experience with a feeling of freedom and ‘*a better experience of my sexual life’,* dedication to their projects, upgrading identity and being happier. This result is not negligible when considering the high suicide rate among MSM [34, 35] (‘*the first representation that I had of homosexuality was linked to death’,’I rediscovered a part of my sexuality, which changed my life’* E7).

Furthermore, the experience of those interviewed revealed a lack of information from general practitioners and a lack of knowledge of PrEP information campaigns. This deficit of information and understanding [36], the negative image of debauch [37], the stigmatisation of PrEP in the general population and even in the MSM community, explain the delay in PrEP recourse. These findings are confirmed by a recent qualitative study of 33 MSM in Singapore [38], which suggests a need to improve professional awareness of the effectiveness of PrEP and to develop a caring approach. We also consider this to be a priority in the French context.

Finally, the main negative point regarding PrEP is the fear of adverse events. All participants were mindful of adverse events and concerned about potential harm. Both this fear, and that of resistance to PrEP [39] were potential barriers to PrEP initiation [14, 16], and needs to be addressed by professionals [40], or improved sexual health promotion through new interventions such as dating apps and social media [41, 42].

### Strengths and limitations

A phenomenological approach allows for a deep exploration of the lived experience, complementing epidemiological research. The semiopragmatic method reveals the logical structure of the participants' lived experience and allows them to understand complex phenomena such as health behaviours. It is the only qualitative analysis method using a formal principle of ordering.

This method provides rich insights into the experiences of MSM PrEP users in France, and the depth of analysis allows the reader to appreciate the nuances in the lived experiences of PrEP use. The validity of our results rests on the authenticity of the responses, and on the triangulation of the analysis with the participation of two experts in the semio-pragmatic approach, limiting the biases of interpretation and ensuring rigour in the construction of categories of meaning.

The fact that doctors invited patients to participate may have induced a coercion bias.

The main limitation of this study is an insufficient diversity of volunteers in terms of socio-economic status, as most had achieved high education levels and held managerial positions. This can be explained either by the choice of purposive sampling, or by the fact that, at the moment, PrEP is mostly accessed by higher socio-economic persons, more comfortable with their sexual identity or who have easier access to medical care and scientific information. Research with participants from less advantaged backgrounds will be needed to explore PrEP among those with other profiles. Although we have information on the use of cannabis, MDMA, alcohol and tobacco, we lack information on specific behaviours (injecting drug user, chemical sex parties and issues of consent when using drugs) in our sample. Peyrières et al. shows a high prevalence of these behaviours by MSM [43].

## Conclusions

This study provides a better understanding of the motivations to engage in PrEP among MSM. Firstly, the persistent fear of HIV infection despite its current representation as a chronic disease; secondly, MSM find that PrEP facilitates a personalised regular follow-up motivating them to take better care of their overall and sexual health; and finally, PrEP changes their lives, allowing them to better live their sexuality through a significant decrease in anxiety and doubt.

## Data Availability

The datasets used and analyzed during the current study are available from the corresponding author on reasonable request.

## Abbreviations

HIV: Human immunodeficiency virus
MSM: Men who have sex with men
PrEP: Pre-exposure prophylaxis
STI: Sexually transmitted infection
